# Using the Internet to Help With Diet, Weight, and Physical Activity: Results From the Health Information National Trends Survey (HINTS)

**DOI:** 10.2196/jmir.2612

**Published:** 2013-08-01

**Authors:** Scout N McCully, Brian P Don, John A Updegraff

**Affiliations:** ^1^Department of PsychologyKent State UniversityKent, OHUnited States

**Keywords:** physical activity, weight loss, dietary habits, Web-based, Internet, utilization

## Abstract

**Background:**

The Internet offers a viable platform for cost-effective and wide-reaching health interventions. However, little is known about use of the Internet to help with diet, weight, and physical activity (DWPA) using a nationally representative sample from the United States.

**Objective:**

To (1) assess the demographic characteristics of people who use the Internet to help with DWPA, (2) assess whether usage trends changed over time, and (3) investigate the associations between using the Internet for DWPA and health behaviors.

**Methods:**

Data on Internet users from the 2007 and 2011 iterations of the Health Information National Trends Survey (HINTS), N=4827 were analyzed using multiple logistic regression to determine the demographic correlates of using the Internet for help with DWPA. Multiple linear regression was used to test the associations between Internet use for DWPA and three health behaviors: fruit intake, vegetable intake, and physical activity.

**Results:**

A larger percentage of Internet users used the Internet for DWPA in 2011 (42.83%) than in 2007 (40.43%). In general, Internet users who were younger (OR 0.98, *P*<.001), more educated (OR 1.40, *P*<.001), married (OR 1.06, *P*=.03), of a minority race (non-Hispanic blacks: OR 1.14, *P*=.02; Hispanics: OR 1.42, *P*=.01), and who had a higher Body Mass Index (BMI) (OR 1.04, *P*<.001) were more likely to use the Internet for DWPA. Across survey years, gender was not associated with using the Internet for DWPA (OR 1.03, *P*=.12), but there was a significant interaction between survey year and gender (OR 1.95, *P*=.002); in 2007, men were more likely to use the Internet for DWPA, but women were more likely to do so in 2011. Using the Internet for DWPA was associated with more vegetable intake (B=.22, *P*=.002), more fruit intake (B=.19, *P*=.001), and more moderate exercise (B=.25, *P*=.001), although the strength of the associations between using the Internet for DWPA and fruit intake and exercise was weaker in 2011 than in 2007.

**Conclusions:**

Contrary to prior research, our population-level study did not show a pronounced gender difference in the use of the Internet for DWPA. Our results support the increasing viability of the Internet as a platform for behavior change intervention, as a growing percentage of Internet users are turning to the Internet for help with DWPA. Additionally, using the Internet for DWPA is associated with better DWPA-related health behaviors.

## Introduction

Poor nutrition and physical inactivity have significant and negative implications for individuals and society at large. On the individual level, poor nutrition and physical inactivity are risk factors for obesity, type 2 diabetes, and heart disease [1-4]. On the societal level, unhealthy lifestyles create a heavy economic burden through largely preventable diseases [5,6]. Thus, focus has turned to prevention, such as modification of behavioral risk factors to reduce incidence of disease. Many evidence-based clinical interventions have been developed to help people manage their diet, weight, and physical activity (DWPA), and a plethora of open-access and/or commercial DWPA programs are available via the Internet [7-9]. Internet-delivered programs are unique in their ability to cost-effectively reach large numbers of users across geographically dispersed areas, provide anonymity for users who wish it, and reduce time and travel demands that in-person programs necessitate [10,11]. Despite the burgeoning availability of DWPA programs, few studies have examined their use at a population level.

The demographic trends in DWPA use provide essential information to those seeking to develop, implement, and evaluate Internet-based DWPA programs. Demographic information provides a basis for tailored and targeted programs, which produce better health outcomes than nontailored or nontargeted programs (see [12] for review). Yet little is currently known about who uses Internet-based DWPA programs because extant usership statistics are based on self-selected samples from randomized clinical trials (RCTs) and certain commercially offered or open-access programs [13-16]. Existing studies suggest that those who elect to participate in Internet-based RCTs for diet and weight loss are primarily female, middle aged, and college educated [13-15]. Specific commercially offered or open-access programs for DWPA indicate a similar pattern; for example, a study of the commercial Web-based weight loss program, *The Biggest Loser Club, Australia,* revealed that 86% of the nearly 10,000 enrollees were female, the majority was of moderate to high socioeconomic status, and the mean age was 35.7 years [16]. Yet, because these statistics describe samples of individuals openly seeking enrollment in an official trial or open-access weight loss program, they may not reflect the average demographics of ad libitum usership of the Internet for DWPA, as only a minority of people who visit a website with an RCT enrollment opportunity elect to enroll [15]. Thus, examining self-selected enrollers in clinical trials or users of a select few open-access and commercial programs may not provide complete demographic data of national usage. Therefore, the first aim of the current study is to document the demographic profile of those who use the Internet to help with DWPA using a nationally representative sample.

Regardless of who uses Internet-based DWPA programs, the viability of the Internet as a platform for wide-reaching health behavior interventions is dependent on these programs actually reaching a large population of users. However, because no large nationally representative studies on trends in Internet use for DWPA have been conducted, it is not known whether Internet use for DWPA is increasing or decreasing. Therefore, the second aim of our study is to investigate temporal trends in usership—namely, whether a higher percentage of Internet users used the Internet for DWPA in 2011 than in 2007, and whether there are important demographic differences in usership over time. Research by the National Telecommunications and Information Administration indicates that overall use and access to the Internet is increasing; that is, more people had Internet in their homes in 2010 compared to 2007 and more people had broadband, indicating faster connection speeds [17]. Additionally, Pew Research Center polls indicate that 81% of adults used the Internet in 2012 compared to 71% in 2007 [18]. With a broader base of Internet users in general, examining whether any demographic shifts in users of the Internet for DWPA occurred between 2007 and 2011 is important, as this information may inform prospective tailoring or targeting of future DWPA programs. Thus, we predict that a higher percentage of Internet users would be using the Internet for DWPA in 2011 than in 2007. We also examine any changes over time in the demographics of people who use the Internet for DWPA.

A third aim of our study is to examine how use of the Internet for DWPA relates to adherence to DWPA-related behaviors. To date, no studies have assessed the relationship between ad libitum use of the Internet for DWPA and health behaviors in a nationally representative study. Because the viability of Internet-based programs depends on their actual association with health behaviors, we aim to examine whether use of Internet-based DWPA programs is associated with the key health behaviors of vegetable intake, fruit intake, and exercise. To investigate these relationships, we examined data from the 2007 and 2011 iterations of the US National Cancer Institute’s Health Information National Trends Survey (HINTS).

## Methods

### Data Source

This study used data from two iterations of HINTS, administered in 2007 and 2011. HINTS is a national probability survey of US adults that assesses usage and trends in health information access and understanding. By oversampling high minority areas, HINTS provides greater precision of estimates for minority subpopulations. HINTS has been administered iteratively, and publicly accessible datasets from 2003, 2005, 2007, and 2011 are available at the HINTS website [19], along with general methodological information about the HINTS survey. The 2007 and 2011 iterations both included an item assessing Internet use for DWPA; no prior iterations included this item. In 2007, two methods were used for data collection: a random digit dial telephone survey and a paper and pencil survey. The mailed survey, but not the random digit dial survey, was used because it assessed fruit and vegetable intake on the same scale (cups per day) as the 2011 survey. The 2007 household response rate for mailed surveys was 40.0%, and the 2011 household response rates were 37.9% for the next-birthday selection method (only the adult whose next birthday is soonest completes the survey) and 35.3% for the all adult selection method (all adults in household complete the survey). More information can be found in the respective cycles’ methodology reports [20,21].

### Participants

A total of 3582 individuals completed the mailed survey in the 2007 HINTS, and 3959 individuals completed the 2011 survey. Of all possible participants, those who had missing data on using the Internet for DWPA (32.8%) or key demographic variables (2.5%) were excluded, resulting in a sample size of 4827 in demographic analyses. Those who had data for using the Internet for DWPA but not for health behavior variables were excluded on an analysis-by-analysis basis, with the greatest missing data for physical activity (10.5%), and negligible missingness for vegetable intake (5 participants), and fruit intake (2 participants).

### Measures

#### Demographics

We used participants’ self-reports of age, sex, level of education, and height and weight. We converted height and weight to body mass index (BMI) scores in which higher scores indicate a generally less healthy weight-to-height ratio.

#### Internet Use for DWPA

Participants answered one question, “In the last 12 months, have you used the Internet to: Use a website to help you with your diet, weight, or physical activity?” with a yes/no response.

#### Fruit and Vegetable Intake

Fruit and vegetable intake were each assessed with the question, “About how many cups of fruit [vegetables] (including 100% pure fruit [vegetable] juice) do you eat or drink each day?” Examples of 1 cup of fruits and vegetables were provided, such as “1 small apple” or “3 broccoli spears.” Participants had 7 response options from “none” to “4 cups or more” such that higher scores represent greater intake. Fruit and vegetable intake scores were analyzed separately.

#### Physical Activity

Physical activity was assessed with the question, “In a typical week, how many days do you do any physical activity or exercise of at least moderate intensity, such as brisk walking, bicycling at a regular pace, and swimming at a regular pace?” The 8 response options ranged from “none” to “7 days per week.”

### Statistical Analyses

#### Combining the Datasets

To analyze differences over time, the 2007 and 2011 datasets were combined using methods employed in prior HINTS analyses [22]. We modified the procedure for the 2007 and 2011 datasets, and for 2007, we used the mail-only final and replicate weights to accurately weight the data based on our exclusive use of mail surveys.

#### Analytic Procedure

Multiple logistic regression was used to determine the demographic correlates of usage of the Internet for DWPA and to assess whether changes occurred in these demographic associations over time. Interaction terms between demographic variables and survey year were included in the model to identify significant changes in demographic makeup of users over time. Multiple linear regression, controlling for demographic variables, was used to analyze the strength of the relationship between use of the Internet for DWPA and the health behaviors of fruit and vegetable intake and physical activity. Interaction terms between Internet use for DWPA and survey year were added to the regressions to assess changes across time. Goodness of fit for all logistic models was assessed with the Hosmer-Lemeshow (H) test statistic and Tjur’s [23] coefficient of discrimination (D). A nonsignificant H statistic indicates good fit, or minimal deviation between observed and predicted values. The D ranges from 0 (no discriminatory power) to 1 (perfect discriminatory power) and can be interpreted roughly as the percent shift in predicted versus observed probabilities compared to a null model. All statistical analyses were conducted using Stata 12, and a cutoff of *P*<.05 was used to determine statistical significance for all analyses.

## Results

### Demographic Predictors of Using the Internet for DWPA

Age, sex, level of education, BMI, race/ethnicity, and marital status were examined as demographic predictors of using the Internet for DWPA. Each increase in level of education (OR 1.40, 95% CI 1.38-1.42, *P*<.001) and in BMI (OR 1.04, 95% CI 1.03-1.04, *P*<.001) was associated with a significantly greater likelihood of using the Internet for DWPA, but each additional year of age was associated with significantly lower likelihood of using the Internet for DWPA (OR 0.98, 95% CI 0.97-0.98, *P*<.001). Married individuals were more likely than unmarried individuals to have used the Internet for DWPA (OR 1.06, 95% CI 1.02-1.11, *P*=.03). Both non-Hispanic blacks (OR 1.14, 95% CI 1.06-1.23, *P*=.02) and Hispanics (OR 1.42, 95% CI 1.20-1.68, *P*=.01) were more likely than non-Hispanic whites to have used the Internet for DWPA. Gender was not related to using the Internet for DWPA (OR 1.03, 95% CI 0.98-1.09, *P*=.12). See Tables 1 and 2 for unweighted group sizes, population-weighted percentages, and means and standard deviations. The logistic model provided adequate fit, H=3.87, *P*=.18, D=.065.

**Table 1:** Demographic correlates of using the Internet for help with DWPA in 2007 and 2011.

		Did not use Internet for DWPA				Used Internet for DWPA			
		2007		2011		2007		2011	
		n	%	n	%	n	%	n	%
Total sample		1462	59.6	1457	57.2	961	40.4	947	42.8
**Gender**									
	Male	878	29.9	630	29.2	640	22.7	309	19.0
	Female	584	29.3	827	28.0	321	17.8	638	23.9
**Race/Ethnicity**									
	Non-Hispanic white	1108	47.8	1006	45.6	674	30.3	622	31.2
	Non-Hispanic black	137	5.2	193	6.7	113	4.7	124	4.7
	Hispanic	114	6.8	113	5.6	90	5.1	100	6.3
**Education**									
	Less than High school	60	4.6	58	3.9	22	1.4	28	2.6
	High school graduate	292	14.1	235	11.5	105	5.9	93	5.7
	Some college	509	24.1	491	21.0	334	17.8	282	14.0
	Bachelor’s	359	10.1	378	11.7	303	9.9	346	14.0
	Post bacc.	242	6.8	295	9.2	197	5.4	198	7.5
**Marital status** ^a^									
	Married	848	35.1	794	33.9	564	24.8	495	22.5
	Single	511	24.2	518	23.9	313	16.0	351	19.7

^a^Marital status was collapsed for simplicity into two categories: married (married or living as married) and single (single, divorced, separated, widowed).

**Table 2:** Demographic and health behavior correlates of using the Internet for help with DWPA in 2007 and 2011.

		Did not use Internet for DWPA				Used Internet for DWPA			
		2007		2011		2007		2011	
		Mean	SD	Mean	SD	Mean	SD	Mean	SD
Age		50.30	16.12	52.74	15.32	44.64	13.44	45.72	13.99
BMI		27.32	6.27	27.40	6.28	28.14	6.60	28.51	6.74
**Health behaviors** ^a^									
	Fruit intake	2.42	1.38	2.38	1.36	2.72	1.36	2.54	1.36
	Vegetable intake	2.77	1.31	2.67	1.36	3.03	1.33	2.88	1.32
	Moderate exercise	3.35	1.84	2.68	2.21	3.57	1.75	2.81	2.06

^a^Fruit (N=4816) and vegetable (N=4813) intake reflect cups per day; moderate exercise (N=4062) reflects days per week.

### Changes in Use of the Internet for DWPA

To determine whether more people used the Internet for DWPA in 2011 than in 2007, we conducted a multiple logistic regression including all of the demographic variables (age, gender, BMI, education, race, and marital status) and survey year. There was a trend that Internet users in 2011 were more likely to have used the Internet for DWPA than were Internet users in 2007 (OR 1.05, 95% CI 0.99-1.12, *P*=.07). The logistic model provided adequate fit, H=.46, *P*=.57, D=.065.

To assess whether the makeup of users had changed between 2007 and 2011, we ran separate multiple logistic regressions in which the demographic variables, survey year, and an interaction term between the survey year and the demographic variable of interest were entered. There was no change across years in BMI (OR 1.00, 95% CI 0.99-1.01, *P*=.98). However, significant changes between 2007 and 2011 were found for gender (OR 1.95, 95% CI 1.73-2.19, *P*=.002), age (OR 0.99, 95% CI 0.98-0.99, *P*=.003), education (OR 0.91, 95% CI 0.90-.93, *P*=.001), and marital status (OR 0.76, 95% CI 0.70-0.84, *P*=.007). In other words, users of the Internet for DWPA were younger, less educated, and more likely to be female and single in 2011 than in 2007. Additionally, a lower proportion of non-Hispanic blacks (OR 0.74, 95% CI 0.66-0.83, *P*=.008) and a higher proportion of Hispanics (OR 1.38, 95% CI 1.06-1.81, *P*=.04) used the Internet for DWPA in 2011 than in 2007, compared to non-Hispanic whites. All models fit the data adequately (*P*s>.17), and addition of these interactions improved model fit (D=.074).

### Health Behaviors Associated With Using the Internet for DWPA

In general, people who used the Internet for DWPA in 2011 reported 2.54 cups of fruit intake per day, 2.88 cups of vegetable intake per day, and 2.81 days of exercise per week (see Table 2). Because we analyzed users of the Internet for DWPA only among those without missing data on the key variable, we descriptively checked health behavior means among those who did not use the Internet at all. In general, people who did not use the Internet at all reported 2.27 cups of fruit intake per day, 2.57 cups of vegetable intake per day, and 2.18 days of exercise per week. The lower levels in health behaviors among non-Internet users suggest that our analysis of Internet users was a more stringent test of the relationship between using the Internet for DWPA and health behavior.

We tested the associations between health behaviors and use of the Internet for DWPA using 3 multiple linear regressions, each controlling for all demographic variables (BMI, age, gender, education, race, and marital status) as well as survey year. People who used the Internet for DWPA reported more vegetable intake (beta=.08, B=.22, 95% CI 0.18-0.27, *P*=.002), more fruit intake (beta=.07, B=.19, 95% CI 0.17-0.21, *P*=.001), and more moderate exercise (beta=.06, B=.25, 95% CI 0.22-0.29, *P=*.001) than those who did not use the Internet for DWPA.

Post hoc analyses were conducted to evaluate whether the relationships between health behaviors and using the Internet for DWPA held for minority groups (ie, non-Hispanic blacks and Hispanics). For the most part, the same positive pattern between health behaviors and use of the Internet for DWPA was observed among Hispanics and non-Hispanic blacks. However, among Hispanics, use of the Internet for DWPA was associated with lower exercise (beta=-.05, B=-.20, 95% CI -0.35 to -0.04, *P*=.03); in 2011, Hispanics who used the Internet for DWPA reported 2.49 days of exercise per week. Thus, Internet sites that specifically target Hispanic populations should be mindful of the somewhat lower exercise adherence levels of Hispanics seeking information about DWPA from the Internet.

To test whether the relationships between using the Internet for DWPA and health behaviors changed from 2007 to 2011, we created interaction terms between use of the Internet for DWPA and survey year, and added the interaction terms to the regressions described above. The interaction term was not associated with vegetable intake (beta=.001, B=.004, 95% CI -0.08 to 0.09, *P*=.83) and was negatively associated with fruit intake (beta=-.07, B=-.25, 95% CI -0.32 to -0.19, *P*=.004) and with moderate exercise (beta=-.01, B=-.05, 95% CI -0.10 to 0.00, *P*=.05). Thus, the relationship between using the Internet for DWPA and eating vegetables was similar in 2011 to what it was in 2007, but that the relationships between using the Internet for DWPA and eating fruit or exercising were weaker in 2011 than in 2007.

## Discussion

### Principal Findings

The current study was the first to examine use of Internet-based DWPA programs in a nationally representative sample of the United States. In general, US adults who were younger, more educated, married, with higher BMIs, and nonwhite were more likely to use the Internet to help with DWPA. The findings for education and BMI are in line with findings from RCTs and studies on commercially available or open-access programs [15,16,24]. While the survey showed that younger people were more likely to use the Internet for DWPA than older people, the mean age of users was 45 (compared to nonusers’ mean age of 51), which is also supportive of previous findings. One surprising finding was that women were no more likely than men to use the Internet for DWPA, in contrast to the large percentages of women who enroll in clinical trials and commercially available/open-access websites [14-16,25]. However, changes in demographic characteristics of users changed significantly between 2007 and 2011, with more females, younger adults, and less educated individuals representing a larger proportion of more recent users. Thus, while this gender profile may have changed over time, our results suggest that men use the Internet for DWPA to a greater extent than previous research suggests. Another surprising finding was that non-Hispanic black and Hispanic Internet users were more likely to have used the Internet for DWPA than non-Hispanic whites. The changes over time indicate an increasing proportion of Hispanic users but a decreasing proportion of non-Hispanic black users. Importantly, these findings indicate that the Internet may serve as a useful platform to help address health disparity gaps traditionally found among minority groups. At a broad level, the changes in age, gender, education level, marital status, and race of the typical user point to the dynamic nature of user characteristics, and those who develop and evaluate Internet programs for DWPA should be aware of changes in usership.

A trend indicated that a greater proportion of Internet users used the Internet for DWPA in 2011 than in 2007. This finding provides support for the increasing viability of the Internet as a platform that can reach large numbers of geographically dispersed people. As more people turn to the Internet for help with DWPA, developing and evaluating quality, evidenced-based online programs is of utmost importance.

Importantly, even when controlling for gender, BMI, age, and level of education, people who use the Internet to help with DWPA report greater fruit and vegetable intake and more physical activity than those who do not use the Internet for DWPA. There are a number of possible explanations for this finding. First, it may reflect pre-existing differences in adherence between those who turn to the Internet for DWPA information compared to those who do not. That is, those who use the Internet for DWPA may have greater interest, intent, or motivation towards DWPA than those who do not seek such information. Second, using the Internet for DWPA may help a person better manage healthy behaviors. However, due the correlational nature of the data, we cannot draw conclusions about the nature of the relationship. To address these causal questions, future studies should examine the relationship between using the Internet for DWPA and related health outcomes in a longitudinal, controlled trial.

We also found that despite more people using the Internet for DWPA in 2011 than in 2007, use in 2011 was associated with lower adherence to healthy DWPA behaviors than in 2007. One potential reason for this finding could be the higher availability of online programs and websites for DWPA; in 2007, it may have taken higher levels of motivation or intention to seek out a website or program for DWPA, and this higher level of motivation may also correspond to better health behaviors. With the proliferation of Internet sites providing DWPA-related information, future users of such sites may be expected to have lower levels of health-behavior adherence than observed in the past. Of course, another possibility is that DWPA-related Internet sites are becoming less effective in their provision of information such that their use leads to lower adherence, but the correlational nature of our data cannot address this possibility. As previously mentioned, only longitudinal, controlled trials can assess the effectiveness of current DWPA Internet sites in improving DWPA behavior.

The US Department of Agriculture and Department of Health and Human Services recommend that adults consume at least 2 cups of fruit and 2.5 cups of vegetables per day given a standard 2000-calorie diet and 150 minutes of moderate physical activity per week [26]. The average self-reported behavior of people who used the Internet for DWPA in 2011 exceeded the recommendations for both fruit and vegetable consumption but likely fell below the recommendations for physical activity. Thus, it could be suggested that people who currently use the Internet for DWPA use it as a support for generally healthy dietary habits, but not as a tool for improving levels of physical activity.

### Limitations

Although this study was the first to assess user characteristics and associated behaviors of using the Internet for DWPA with a nationally representative sample, it is not without limitations. One limitation was the dichotomous nature of our measure for using the Internet for DWPA; there was no way to ascertain an individual’s level of use (eg, yearly, weekly, daily), nor to tell the type or quality of the website or program they used. Second, because the 2007 and 2011 HINTS datasets used different samples, we could not make any longitudinal inferences from the data. Last, all data are self-reported, so our data on DWPA-related behaviors cannot be validated against objective measures.

### Conclusion

The current study advances prior research by examining the demographic and health behavior correlates of Internet use for DWPA among a nationally representative US sample. As Internet use grows to near ubiquity in developed countries, our findings highlight the importance of evaluating Web-based programs, which may prove effective in health behavior change. The Internet represents a viable platform for targeting health behavior change across a large and growing audience, and future research should continue to explore this important topic.

## Abbreviations

DWPA: diet, weight, and physical activity
HINTS: Health Information National Trends Survey
RCT: randomized controlled trial
